# Implementation of Circular Economy Principles in the Synthesis of Polyurethane Foams

**DOI:** 10.3390/polym12092068

**Published:** 2020-09-12

**Authors:** Maria Kurańska, Milena Leszczyńska, Elżbieta Malewska, Aleksander Prociak, Joanna Ryszkowska

**Affiliations:** 1 Department of Chemistry and Technology of Polymers, Cracow University of Technology, Warszawska 24, 31-155 Kraków, Poland; elzbieta.malewska@pk.edu.pl (E.M.); aleksander.prociak@pk.edu.pl (A.P.); 2 Faculty of Materials Science and Engineering, Warsaw University of Technology, Wołoska 141, 0-507 Warsaw, Poland; milena.leszczynska.dokt@pw.edu.pl (M.L.); joanna.ryszkowska@pw.edu.pl (J.R.)

**Keywords:** polyurethane bio-foams, used cooking oil-based polyol, low-density materials

## Abstract

The main strategy of the European Commission in the field of the building industry assumes a reduction of greenhouse gas emissions by up to 20% by 2020 and by up to 80% by 2050. In order to meet these conditions, it is necessary to develop not only efficient thermal insulation materials, but also more environmentally friendly ones. This paper describes an experiment in which two types of bio-polyols were obtained using transesterification of used cooking oil with triethanolamine (UCO_TEA) and diethylene glycol (UCO_DEG). The bio-polyols were next used to prepare low-density rigid polyurethane (PUR) foams. It was found that the bio-polyols increased the reactivity of the PUR systems, regardless of their chemical structures. The reactivity of the system modified with 60% of the diethylene glycol-based bio-polyol was higher than in the case of the reference system. The bio-foams exhibited apparent densities of 41–45 kg/m^3^, homogeneous cellular structures and advantageous values of the coefficient of thermal conductivity. It was observed that the higher functionality of bio-polyol UCO_TEA compared with UCO_DEG had a beneficial effect on the mechanical and thermal properties of the bio-foams. The most promising results were obtained in the case of the foams modified in 60% with the bio-polyol based on triethanoloamine. In conclusion, this approach, utilizing used cooking oil in the synthesis of high-value thermal insulating materials, provides a sustainable municipal waste recycling solution.

## 1. Introduction

Rigid polyurethane (PUR) foams with closed cell structures are used in various branches of industry [1,2]. Most of the industrial halls made of sandwich panels have polyurethane foams inside. Rigid polyurethane foams also insulate refrigerators and pipelines, fill bumpers in cars and are used in aircraft insulation. PUR foams are also used to isolate hives. Such a broad area of applications causes that activities of researchers in the field of the polyurethane technology are focused on looking for new renewable sources of raw materials for their preparation.

Nowadays, the polyurethane industry is dependent on petroleum because a majority of the components used in their preparation are petroleum-based. In order to reduce the consumption of petrochemical components in the synthesis of polyurethanes, both plant and waste fillers are also introduced. The literature describes the influence of such waste as rapeseed cake [3], basalt powder [4], thermoset polyester-glass fiber composite [5], eggshells [6], wheat straw lignin [7], brewers’ spent grain and ground tire rubber [8], buffing dust generated in leather industry [9] on the properties of polyurethane foams. Most results confirm that waste fillers can be successfully used as low-cost and environmentally friendly components for PUR foam modification.

Polyurethanes can be modified by replacing, completely or partially, polyols, which are one of two main components in their synthesis. Bio-polyols are obtained both from vegetable oils (rapeseed, palm, soybean) and from used cooking oils as waste materials [10]. Application of rapeseed oil in polyol synthesis, compared to petrochemical polyols, has a significant impact on the reduction of non-renewable energy use, lower greenhouse gas emissions and water consumption [11]. It can be expected that utilization of used vegetable oil will have an even better effect on the environment in terms of waste management. This idea is in line with new circular economy-related trends to find alternative components for the polymer synthesis based on waste [12]. Zhang et al. [13] prepared four sets of bio-based foams using bio-polyol based on agricultural wastes (oilseed rape straw, rice straw, wheat straw and corn stover). They concluded that the bio-foams with an appropriate NCO/OH group ratio exhibited excellent morphological, physical and mechanical properties, which are comparable with or even better than those of petroleum polyol-based PU foams.

Borowicz et al. carried out glycerolysis of waste poly(lactic acid) in order to obtain oligomeric polyhydric alcohols. Authors concluded that the chemical structure and other physicochemical properties of the final polyols indicated that they could be an alternative to petrochemical polyols [14]. Modification of lignin, a by-product in the pulp and paper industry, has also been described in several studies concerning the synthesis of polyols and their use in polyurethane formulations [15,16]. Hejna et al. obtained bio-polyol via crude glycerol polymerization and further condensation of the resulting polyglycerol with castor oil [17]. In previous works of the authors, it was reported that used cooking oil can be a source of raw materials for bio-polyol synthesis. Ten different samples of used cooking oil were collected from local restaurants and their properties were evaluated. It was concluded that the properties of the bio-components based on used cooking oils were comparable to the properties of the epoxidized oil and polyols obtained from the fresh rapeseed oil that was used as a reference sample [12]. In the literature, there are no reports concerning the influence of bio-polyols obtained through transesterification of used cooking oil with triethanolamine and diethylene glycol on the reactivity of PUR systems and the properties of bio-foams. 

In this study, two types of bio-polyols synthesized by transesterification of used cooking oil with diethylene glycol and triethanolamine were used to prepare bio-based PUR foams with different contents of the bio-components. To obtain PUR foams with excellent properties, detailed analyses were conducted of the foaming process, density, compression strength and coefficient of thermal conductivity. Scanning electron microscope and thermogravimetric analyses were also carried out. The bio-based foams were compared with a petroleum polyol-based foam to confirm their substitutability. The development of an innovative material based on modified municipal waste is a significant step towards implementation of circular economy in the polyurethane industry.

## 2. Materials and Methods 

Rigid PUR foams were prepared using polyol Rokopol RF551 (oxypropylenated sorbitol, PCC Rokita, Brzeg Dolny, Poland), which was partially replaced by bio-polyols based on waste oil (UCO_TEA or UCO_DEG). Bio-polyols were synthesized using the transesterification method of waste oil with triethanolamine (UCO_TEA) and diethylene glycol (UCO_DEG). The molar ratio of the reagents was 1:3 (waste oil:transesteryfication agent). The reaction was conducted at 175 °C and the content of the catalyst (zinc acetate) was 0.3 wt.%. Characteristics of the bio-polyols and Rokopol RF551 are shown in Table 1. 

**Figure 1:**
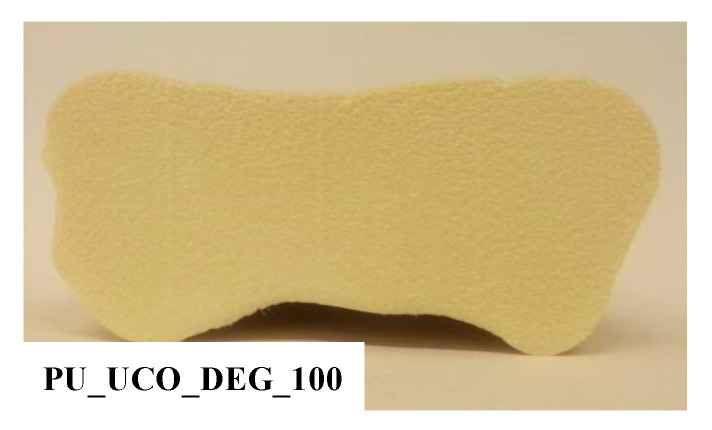
Foam PU_UCO_DEG_100 characterized by shrinkage.

Polymeric diphenylmethane-4,4′-diisocyanate (PMDI) (Ongronat 2100, Borschodchem, Berente, Hungary) with a 31% content of NCO was used as an isocyanate component. An NCO index of 110 was applied in all foams. An amine catalyst (Polycat^®^ 9, Evonik Nutrition & Care GmbH, Essen, Germany) and silicone surfactant (Niax Silicone L-6900, Momentive Performance Materials, Wilton, CT, USA) were used as additives. The components (except PMDI) were mixed for 30 s and then the right PMDI amount was added and mixed for 10 s. The foams were prepared in open molds. The rigid PUR formulations are given in Table 2. All foams were conditioned at room temperature for 24 h. The compositions had 20%, 40%, 60% and 80% of bio-polyol. The materials obtained with a 100% addition of bio-polyol were characterized by shrinkage (Figure 1), and, therefore, could not be subjected to further analysis of physico-mechanical properties. A reference material based on the petrochemical polyol Rokopol RF551 was also obtained.

The foaming process was analyzed using a FOAMAT device (Messtechnik, Freiburg im Breisgau, Germany) that allows determination of e.g., the reaction temperature and dielectric polarization during a foaming process. The apparent density (kg/m^3^) was determined according to ISO 845. The closed-cell content (%) was determined according to ISO 4590.

The foam cell morphology was analyzed using a Hitachi TM3000 scanning electron microscope (Tokyo, Japan) with an accelerating voltage of 5 keV using orientations parallel and perpendicular to the foam growth direction. Prior to the analysis the samples were dusted with gold using a Polaron SC7640 sputter coater for 100 s at 10 mA. The equivalent pore diameter and anisotropy coefficient were determined based on 200 pore dimensions for each material, using SEM images of the samples analyzed in the cross-sections parallel to foam growth direction. The analysis was done using ImageJ software.

The coefficient of thermal conductivity (mW/m·K) was determined using a Laser Comp heat flow instrument Fox 200 (New Castle, DE, USA). The measurements were taken at an average temperature of 10 °C. A compression test was carried out according to ISO844. The analysis was done in a parallel and perpendicular direction to the foam rise.

Infrared spectra of the foams were recorded using a Nicolet 6700 (Thermo Electron Corporation, Madison, WI, USA) FTIR spectrophotometer. Each sample was scanned 64 times in 4000–400 cm^−1^ range of wavenumbers. Data processing was performed using Omnic Spectra 8.2.0 software developed by Thermo.

In order to determine the course of the thermal degradation of the foams, a thermogravimetric analysis was conducted using a TA Instruments Q500 (New Castle, Delaware, DE, USA). Samples with a weight of 10 ± 0.2 mg were placed on platinum pans and heated in a nitrogen atmosphere at 10 °C/min in a temperature range of 25–1000 °C. Data analysis was done with Universal Analysis 2000 software, version 4.7 A, by TA Instruments.

The phase transition temperatures and thermal effects were determined using a differential scanning calorimeter Q1000 (TA Instruments). The measurements were taken in a neutral gas atmosphere using hermetic aluminum cups. The samples (5 ± 0.2 mg) were heated at 10 °C/min in the temperature range from −90 to 200 °C. Data analysis was performed using Universal Analysis 2000 software, version 4.7 A, by TA Instruments.

## 3. Results

**Figure 2:**
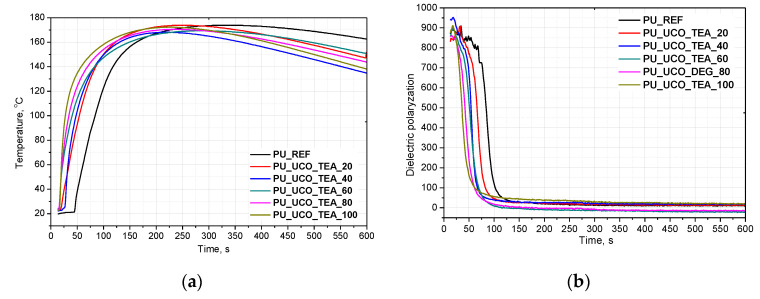
Foams core temperature profiles (**a**) and dielectric polarization changes in time (**b**) during foaming process of PUR system based on UCO_TEA.

**Figure 3:**
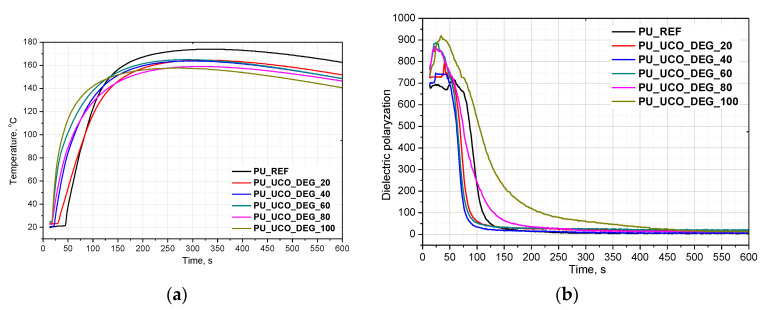
Foams core temperature profiles (**a**) and dielectric polarization changes in time (**b**) during foaming process of PUR system based on UCO_DEG.

The investigation was carried out to determine the influence of different contents of the bio-polyols in the PUR formulation on the foaming process, mechanical properties and structure of rigid foams. Changes in temperature recorded by Foamat are shown in Figure 2a and Figure 3a. The reactivity of a PUR system can be determined by measuring the dielectric polarization, which decreases as an effect of the reactions progress. Figure 2b and Figure 3b show the dielectric polarization as a function of the reaction time for all the samples prepared with UCO_TEA and UCO_DEG, respectively.

As can be seen in Figure 2a and Figure 3a, the bio-based polyurethanes modified with the bio-polyol are characterized by the fastest increase of temperature indicating a greater reaction heat release and a higher extent of the isocyanate–polyol reaction. The higher the content of the bio-polyols in the polyol premix, the faster the temperature increase in the foam core. This effect is similar for the systems based on UCO_DEG as well as UCO_TEA. However, in the case of the systems based on UCO_DEG, the maximum temperature is lower than in the systems modified with the bio-polyol UCO_TEA. Such an effect is favorable in order to avoid scorch while seasoning foam blocks. Based on the faster increase of the dielectric polarization, it was concluded that the PUR systems modified with bio-polyol UCO_TEA were characterized by higher reactivity than the reference system [18]. A replacement of petrochemical polyol with UCO_DEG of up to 60% mas. also caused an increase in the PUR system reactivity. The higher reactivity can be associated with lower viscosity of the bio-polyols and better accessibility to functional groups. Additionally, the bio-polyol UCO_TEA has a catalytic effect on the polyurethane forming reaction due to triethanolamine, which is used in the synthesis of this bio-polyol. Therefore, the more UCO_TEA used, the faster the reaction of forming polyurethane bonds and the faster the temperature increase in this system. In the case of the use of UCO_DEG, it was observed that the dielectric polarization for the systems containing 20%, 40% and 60% of that bio-polyol was similar to that of the reference system. However, the addition of 80% and 100% of this bio-polyol caused that the dielectric polarization curve approached zero slower, which means that the reaction of polyurethane formation in these systems was progressing slower. DEG does not have catalyzing properties as it is in the case of TEA. Additionally, UCO_DEG has three times lower functionality in relation to petrochemical polyol, which also could have affected the process.

**Figure 4:**
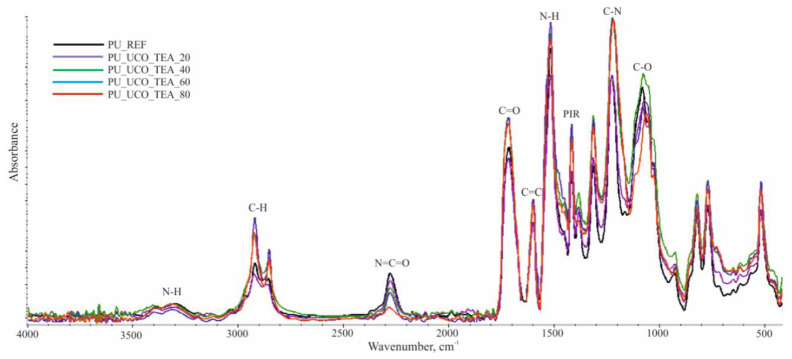
FTIR spectra of the reference material and PU_UCO_TEA_20–80 foams.

The FTIR spectra indicate the presence of groups characteristic for polyurethanes, which confirms the correct course of the reactions. Example FTIR spectra are shown in Figure 4.

The presence of the -N–H groups is indicated by the band at the 3400–3200 cm⁻^1^ wavenumber range (N–H stretching vibrations, symmetric and asymmetric) and the signal with a maximum at 1512–1511 cm⁻^1^ (N–H deformation vibrations). The bands with maxima at 2922–2917 cm⁻^1^ and 2853–2851 cm⁻^1^ correspond to the asymmetric and symmetric stretching vibrations of the C–H bonds in the -CH_3_ and -CH_2_- groups. An analysis of the reference material revealed an additional signal at 2971 cm⁻^1^, originating from the asymmetrical stretching vibrations of the C–H bonds, which is associated with the chemical structure of polyol Rokopol RF551. A low-intensity peak at 2277–2274 cm⁻^1^ corresponding to unreacted isocyanate moieties was also observed, which is a consequence of an isocyanate index of more than 100. The signals with a maxima at 1713–1709 cm⁻^1^ indicate the presence of the C=O carbonyl bonds in urethane groups, while the signal at 1595 cm^−1^ represents the aromatic rings in the material. The signals at 1411 cm⁻^1^ indicate the presence of the isocyanate trimerization products. The presence of isocyanurate rings in the foam is also evidenced by vibration bands of the –NH- and C=O groups at 780 and 525 cm⁻^1^, respectively. The signals with maxima at 1218–1214 cm⁻^1^ correspond to the C-N groups stretching vibrations. The multiplet bands in the range of 1250–1000 cm⁻^1^ are assigned to the C–O bonds in flexible segments. The signals at 2275–2277 cm⁻^1^ correspond to the N=C=O groups. In Figure 4, it may be noticed that the higher the content of the bio-polyol, the lower the intensity of the signal is. This effect can be associated with higher reactivity of the bio-polyols, which is confirmed by the changes of dielectric polarization (Figure 2b). 

**Figure 5:**
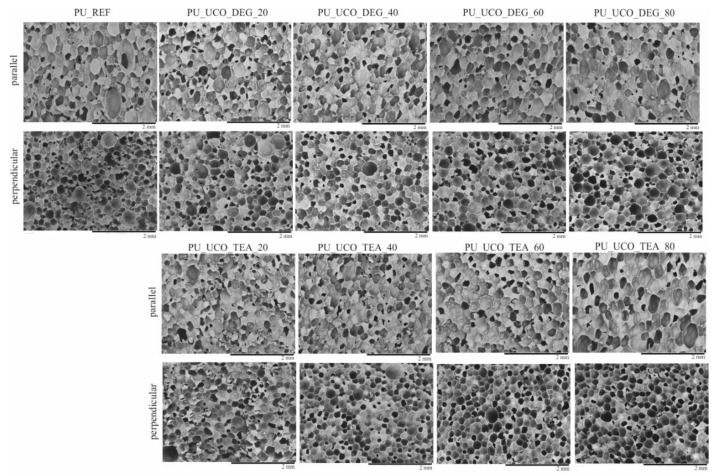
SEM images of polyurethane foams.

Cellular structure has a significant influence on the physical and mechanical properties of porous materials and thus is one of their most important properties [3]. The cell structure parameters such as cell size and cell type (closed or open) depend on the foaming process. SEM microphotographs, the equivalent diameter and anisotropy index of the reference foam and the foams modified with two types of bio-polyols are shown in Figure 5 and Table 3.

**Figure 6:**
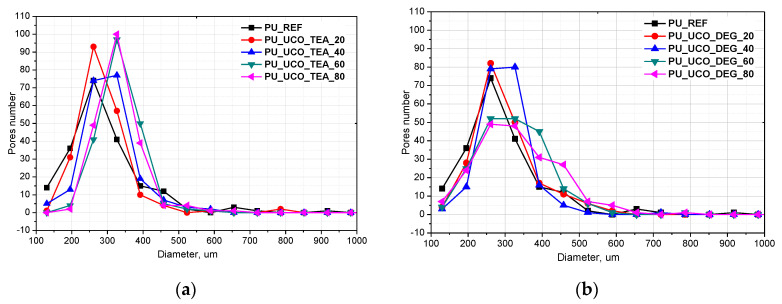
Pore size distribution for PU_REF and foams modified with polyols UCO_TEA (**a**) and UCO_DEG (**b**).

The use of the bio-polyols resulted in the formation of a foam structure with a more regular pore distribution compared to the reference material. Moreover, the introduction of the bio-polyols into the composition yielded foams with a lower pore anisotropy index. The pore size distribution curves for the PU_REF, PU_UCO_DEG_20 and PU_UCO_DEG_40 materials are similar, but the standard deviation of the average diameter equivalent for the reference foam is higher due to the presence of large pores with sizes > 600 µm. Increasing the UCO_DEG polyol content further led to an increase in the average pore diameter and an increase in the pore size range. The pore size of the foams made using UCO_TEA increased after the introduction of 40%–80% of the bio-polyol. The cell structure of the materials made using UCO_TEA was more regular compared to the foams obtained using UCO_DEG, which is indicated by the lower value of the standard deviation of the equivalent diameter for PU_60_TEA and PU_80_TEA foams compared to the PU_60_DEG and PU_80_DEG foams. The foams with a high proportion of the bio-polyol from the TEA series (60%–80%) have a lower anisotropy index compared to the DEG series. The lower anisotropy index and more regular structure of the foams based on the bio-polyol UCO_TEA can be associated with the higher reactivity of the foams modified with 60% and 80% of this bio-polyol. Such a structure had a beneficial effect on the thermal conductivity and mechanical properties of the foams based on the bio-polyol UCO_TEA (Table 4 and Figure 6).

The foam materials were subjected to an analysis of physico-mechanical properties, i.e., apparent density, thermal conductivity, closed cell content, compressive strength at 10% deformation in a perpendicular and parallel directions to the direction of foam growth. The results are presented in Table 4 and Figure 6.

The foam materials obtained with the use of UCO_TEA and UCO_DEG were characterized by a lower apparent density in relation to the reference material. In addition, it was observed that for most materials the higher the content of the bio-polyol used in the formulation, the lower the apparent density. This is due to lower viscosity of the bio-polyols which facilitated the growth of the foam material and greater system reactivity, especially in the case of UCO_TEA modified foams. In most of the materials obtained, the thermal conductivity was comparable or slightly higher than in the case of the reference material. Generally, the thermal conductivity of PUR foams is dependent on the thermal conductivity of the solid phase, gas enclosed in cells as well as convective and radiative components [19]. The foams prepared in this work had a low apparent density, in which the solid polymer phase constituted only ca. 3% of the foam, and, therefore, its contribution to the overall foam conductivity can be neglected. The value of the thermal conductivity is greatly influenced by the closed cell content and cell size of foams. In the case of PU_UCO_DEG_60 foam, the cells were partially open, which resulted in an increase in the heat transfer coefficient leading to thermal conductivity value about 34 mW/m·K. Such an effect may be related to insufficient miscibility of the polyols. In the systems containing 40% and 60% of the bio-polyol, the bio-polyol does not dominate, hence the lower miscibility. However, in the case of the PU_UCO_DEG_40 system, this effect does not occur, which may be related to much higher functionality of the petrochemical polyol and a higher tendency of the system for crosslinking.

The compressive strength of porous polyurethanes depends on foam morphologies (cell size and content of closed cells), foam density and polyol functionality [19]. A high value of compressive strength at a low apparent density of foams results from a small cell size and high functionality. In this experiment, bio-polyols UCO_DEG and UCO_TEA were characterized by much lower functionality than Rokopol RF551, and an analysis of the compressive strength changes in relation to the bio-polyol content was necessary.

**Figure 7:**
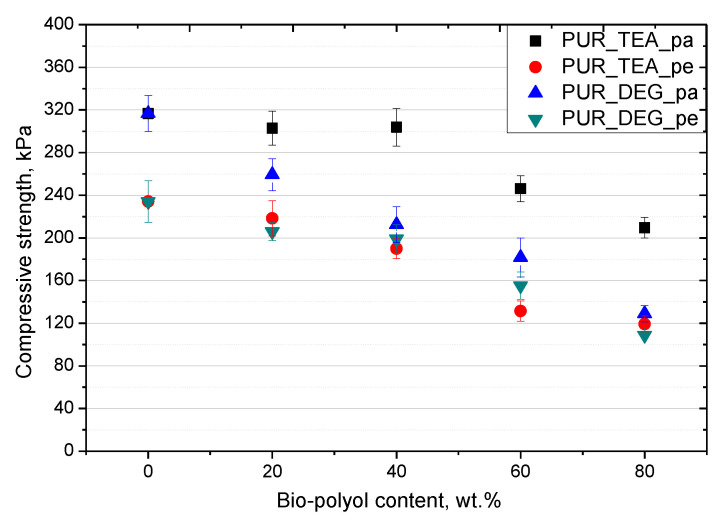
Compressive strength parallel (pa) and perpendicular (pe) to direction of foam rise of rigid PUR materials containing UCO_TEA and UCO_DEG bio-polyols.

The analysis of the compressive strength of the foam materials was carried out in a perpendicular and parallel direction to the direction of growth. The results are shown in Figure 7. It was observed that in all the materials the compressive strength in the parallel direction was greater than in the perpendicular direction. It is related to the shape of the cells that were formed [9]. Cells were usually elongated in the growth direction of a material, which promotes a higher value of the compressive strength. It was also observed that the compressive strength decreased with an increasing bio-polyol content in the perpendicular and parallel direction to the foam growth direction for both the bio-polyols used. The foams containing 20% and 40% of UCO_TEA had a strength similar to the reference material, when it was measured in the direction parallel to the direction of foam growth. As the bio-polyol content increased over 40%, the compressive strength of the modified foams decreased for both the UCO_TEA and UCO_DEG bio-polyols.

That was a result of the PUR matrix plasticizing by the dangling chains of fatty acids of the bio-polyols [19]. However, the mechanical strength was at an appropriate level to ensure the dimensional stability of the foams. The higher compressive strength of the foams modified with UCO_TEA is an effect of the higher functionality of this bio-polyol (Table 1). 

**Figure 8:**
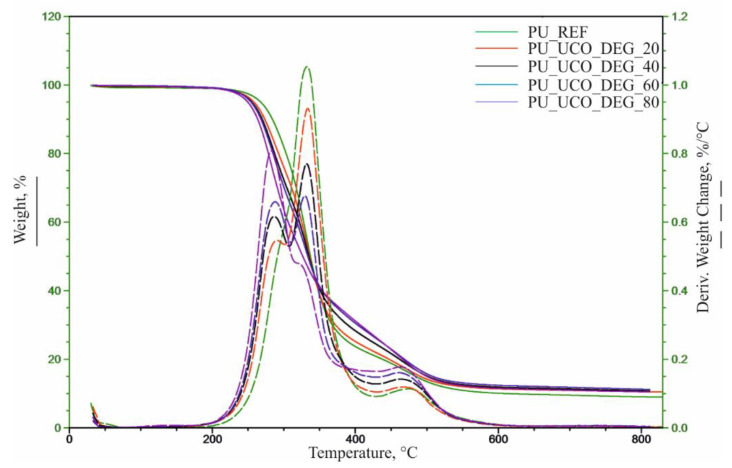
Thermograms for reference material and PU_UCO_DEG foams.

**Figure 9:**
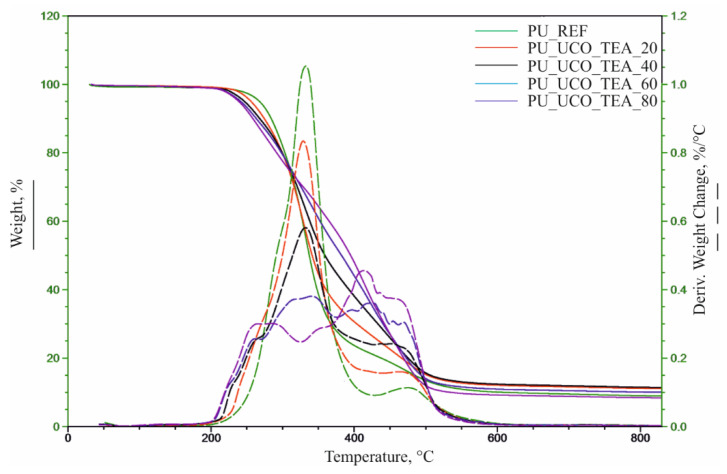
Thermograms for reference material and PU_UCO_TEA foams.

Based on the TG and DTG curves, the following parameters were determined:, temperatures at 5%, 10%, 50% and 70% weight loss of the materials (T_5%_, T_10%_, T_50%_, T_75%_), temperatures at the maximum degradation rates (T_max_) and the corresponding degradation rates (V_max_) in subsequent thermal decomposition stages (Table 5, Figure 8 and Figure 9). 

The results of the analysis indicate that the introduction of the bio-polyols from used cooking oils caused a decrease in the temperature at the beginning of the foam thermal degradation (T_5%_) compared to the reference material. The foams based on the UCO_TEA polyol exhibited a significantly higher temperature reduction in comparison with the foams based on the UCO_DEG bio-polyol. Based on the DTG curves, it was found that the thermal degradation of the reference material occurred in two stages in the range of 200–420 and 420–550 °C. The first stage (T_max2_, V_max2_) is associated with the degradation of the mixture of flexible and rigid segments, and the second stage (T_max3_, V_max3_) is associated with thermolysis of organic residues [20,21]. The use of the UCO_DEG and UCO_TEA bio-polyols for the production of foams led to changes in the foam structure and, consequently, in the course of the DTG curve. The introduction of UCO_DEG in the amount of 20%–80% and UCO_TEA in the amount of 40%–80% resulted in the observation of an additional stage in the thermal decomposition pathway corresponding to the degradation of rigid segments (T_max1_, V_max1_). The content of the bio-polyols was correlated with the rate of foam degradation at this stage (V_max1_), consequently decreasing the temperature of 10% weight loss of the material. The changes may be related to the increased degree of phase separation in the foams containing the bio-polyols. The use of high contents of the UCO_TEA polyol drastically changed the course of the foam thermal degradation pathway from a well-resolved 3-stage case to a complex process involving a multitude of overlapping steps spanning in the range of 280–500 °C. The results of the analysis indicate a higher thermal stability of the segments formed from the UCO_TEA bio-polyol, which is confirmed by an increase of the 50% and 75% mass loss temperature and an increase of the V_max_ value of the last degradation stage. The foams prepared from the UCO_DEG bio-polyol degraded in 50% at comparable temperature values to the reference material and exhibited a slowdown in the further degradation stage, which is indicated by an increase in the 75% mass loss temperature of the material and an increase in the V_max3_ value. The percentage of combustion residue at 600 °C (R_600_) slightly increases after the introduction of the bio-polyols.

**Figure 10:**
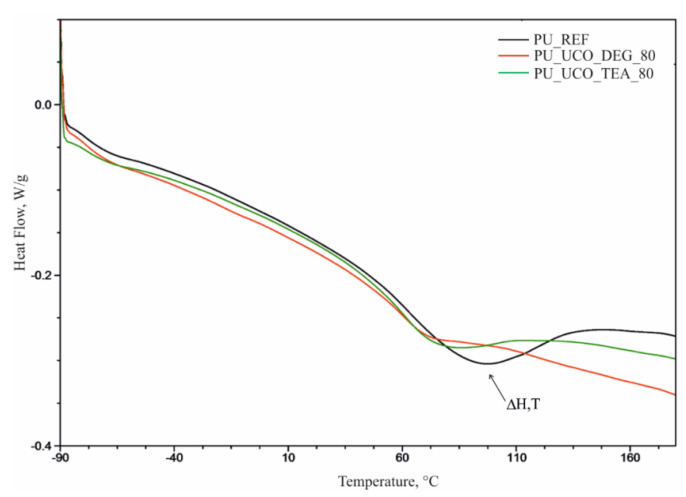
DSC thermograms of materials.

The DSC thermograms (Figure 10) display endothermic peaks in the range of −20–170 °C corresponding to the order-disorder transformation in polyurethane foams. The replacement of the petrochemical polyol with either bio-polyol in the foam resulted in a decrease of the enthalpy of this transformation (ΔH). The thermal effect of this transformation is higher for the TEA series than for the DEG series (Table 6). 

The results of the extreme phase transition temperature (T) indicate that it decreases as the content of the bio-polyol increases. The cause is the plasticizing effect of the unsaturated fatty acid hydrocarbon chains found in natural oil. Example DSC thermograms for PU_REF, PU_UCO_DEG_80 and PU_UCO_TEA_80 materials are shown in the Figure 10.

## 4. Conclusions

Used cooking oil was converted into two bio-polyols using the transesterification method. Two different transesterification agents were used in the reactions—triethanolamine and diethylene glycol. The bio-polyol based on triethanolamine was characterized by higher reactivity than the one based on diethylene glycol. It was observed that the PUR systems modified by up to 60% with the bio-polyol based on diethylene glycol had higher reactivity than the reference system. Such an effect has not been described in the literature so far. This polyol property allows for decreasing the content of catalysts in a PUR system. The bio-foams modified with the triethanoloamine-based bio-polyol were characterized by a higher compressive strength parallel to the foam rise direction regardless of the bio-polyol content due to higher functionality of this bio-component comparing to the diethylene glycol based bio-polyol. The most promising results were obtained in the case of the foams modified in 60% with the bio-polyol based on triethanoloamine. The use of waste oil as a renewable resource for the synthesis of bio-polyols as well as the possibility to apply lower catalyst contents allows for implementing selected rules of green chemistry and a circular economy in the synthesis of polyurethane foams.

## Figures and Tables

**Table 1:** Characteristics of polyols.

Properties	UCO_TEA	UCO_DEG	Rokopol RF551
Synthesis method	Transesterification of waste oil with triethanoloamine	Transesterification of waste oil with glycol diethylene	Oxyalkylation of sorbitol
Hydroxyl value, mgKOH/g	348	277	420
Acid value, mgKOH/g	2.31	1.35	0.1
Water content, % mas	0.05	0.13	0.1
Molecular weight, g/mol	522	492	~600
Viscosity, mPa·s	182	56	3000–5000
Functionality	~2.2	~1.9	~4.5

**Table 2:** Formulations of foam materials obtained with different amounts of the bio-polyols.

Foam Symbol	Component (g)					
	Petrochemical Polyol	Bio-Polyol	Catalyst	Silicone Surfactant	Water	PMDI
PU_REF	100	0	1.50	1.50	3.00	165.8
PU_UCO_TEA_20	80	20	1.50	1.50	3.00	160.6
PU_UCO_TEA_40	60	40	1.50	1.50	3.00	155.5
PU_UCO_TEA_60	40	60	1.50	1.50	3.00	150.3
PU_UCO_TEA_80	20	80	1.50	1.50	3.00	145.2
PU_UCO_DEG_20	80	20	1.50	1.50	3.00	155.8
PU_UCO_DEG_40	60	40	1.50	1.50	3.00	145.8
PU_UCO_DEG_60	40	60	1.50	1.50	3.00	135.9
PU_UCO_DEG_80	20	80	1.50	1.50	3.00	125.9

**Table 3:** Characteristic properties of cellular structure of PUR foams.

Symbol	Equivalent Diameter, µm	Anisotropy Index
PU_REF	265 ± 133	1.41 ± 0.23
PU_UCO_TEA_20	256 ± 103	1.34 ± 0.19
PU_UCO_TEA_40	274 ± 75	1.39 ± 0.18
PU_UCO_TEA_60	301 ± 54	1.24 ± 0.13
PU_UCO_TEA_80	300 ± 63	1.26 ± 0.16
PU_UCO_DEG_20	265 ± 85	1.27 ± 0.15
PU_UCO_DEG_40	266 ± 66	1.36 ± 0.19
PU_UCO_DEG_60	290 ± 93	1.38 ± 0.20
PU_UCO_DEG_80	299 ± 109	1.31 ± 0.18

**Table 4:** Apparent density, heat conduction coefficient and closed cell content of foam materials containing different amounts of UCO_TEA and UCO_DEG.

Symbol	Apparent Density, kg/m^3^	Thermal Conductivity, mW/m·K	Closed Cell Content, %
PU_REF	48.4 ± 1.4	25.88 ± 0.62	80.0 ± 1.9
PU_UCO_TEA_20	45.3 ± 0.5	25.49 ± 0.64	81.0 ± 0.1
PU_UCO_TEA_40	43.9 ± 1.0	26.16 ± 0.29	80.3 ± 1.5
PU_UCO_TEA_60	41.2 ± 0.1	25.72 ± 0.77	82.3 ± 1.6
PU_UCO_TEA_80	41.1 ± 0.0	27.40 ± 0.21	83.7 ± 3.0
PU_UCO_DEG_20	43.6 ± 0.6	24.69 ± 0.50	86.1 ± 0.1
PU_UCO_DEG_40	41.7 ± 1.9	28.92 ± 0.87	78.2 ± 0.6
PU_UCO_DEG_60	40.0 ± 0.2	34.03 ± 0.65	26.9 ± 8.8
PU_UCO_DEG_80	41.5 ± 0.0	28.28 ± 0.80	78.0 ± 1.4

**Table 5:** Results of thermogravimetric analysis.

Symbol	T_5%_, °C	T_10%_, °C	T_50%_, °C	T_75%,_ °C	T_max1,_ °C (V_max1,_ %/°C)	T_max2_ (V_max2_), °C	T_max3_ (V_max3_), °C	R600, %
PU_REF	268	285	337	390	-	333 (1.049)	475 (0.096)	10.3
PU_UCO_TEA_20	256	274	341	435	-	329 (0.834)	464 (0.159)	12.1
PU_UCO_TEA_40	247	268	359	454	263 (0.255)	332 (0.581)	454 (0.242)	12.4
PU_UCO_TEA_60	241	263	382	456	261 (0.256)	316 (0.372), 341 (0.381), 397 (0.341), 424 (0.361), 441 (0.333), 454 (0.308), 469 (0.304)		11.1
PU_UCO_TEA_80	239	259	397	457	266 (0.289)	288 (0.290), 351 (0.293), 412 (0.469), 430 (0.409), 446 (0.387), 461 (0.378)		9.7
PU_UCO_DEG_20	258	273	336	404	290 (0.546)	333 (0.931)	467 (0.119)	12.1
PU_UCO_DEG_40	255	270	335	424	287 (0.617)	332 (0.769)	464 (0.142)	11.9
PU_UCO_DEG_60	252	265	334	437	285 (0.664)	328 (0.652)	457 (0.157)	12.3
PU_UCO_DEG_80	250	263	327	437	283 (0.808)	322 (0.477)	468 (0.177)	11.5

**Table 6:** Results of DSC analysis.

Symbol	ΔH, J/g	T, °C
PU_REF	50.3	87.9
PU_20_DEG	27.0	88.3
PU_40_DEG	17.0	77.1
PU_60_DEG	16.9	73.0
PU_80_DEG	11.0	71.1
PU_20_TEA	29.7	83.2
PU_40_TEA	30.3	80.9
PU_60_TEA	24.9	80.5
PU_80_TEA	27.1	75.7

## References

[1] Liszkowska J., Borowicz M., Paciorek-Sadowska J. (2019). Assessment of Photodegradation and Biodegradation of RPU/PIR Foams Modified by Natural Compounds of Plant Origin. Polymers.

[2] Zhang G., Wu Y., Chen W., Han D., Lin X., Xu G. (2019). Open-Cell Rigid Polyurethane Foams from Peanut Shell-Derived Polyols Prepared under Di ff erent Post-Processing Conditions. Polymers.

[3] Paciorek-Sadowska J., Borowicz M., Isbrandt M., Czupryński B., Apiecionek Ł. (2019). The Use of Waste from the Production of Rapeseed Oil for Obtaining of New Polyurethane Composites. Polymers.

[4] Kurańska M., Barczewski M., Uram K., Lewandowski K., Prociak A., Michałowski S. (2019). Basalt waste management in the production of highly effective porous polyurethane composites for thermal insulating applications. Polym. Test..

[5] Barczewski M., Kurańska M., Sałasińska K., Michałowski S., Peociak A., Uram K., Lewandowski K.K. (2019). Rigid polyurethane foams modified with thermoset polyester-glass fiber composite waste. Polym. Test..

[6] Zieleniewska M., Leszczyński M.K., Szczepkowski L., Bryśkiewicz A., Krzyżowska M., Bień K., Ryszkowska J. (2016). Development and applicational evaluation of the rigid polyurethane foam composites with egg shell waste. Polym. Degrad. Stab..

[7] Paberza A., Cabulis U., Arshanitsa A. (2014). Wheat straw lignin as filler for rigid polyurethane foams on the basis of tall oil amide. Polimery.

[8] Formela K., Hejna A., Zedler Ł., Przybysz M., Ryl J., Reza M., Piszczyk Ł. (2017). Structural, thermal and physico-mechanical properties of polyurethane/brewers’ spent grain composite foams modified with ground tire rubber. Ind. Crop. Prod..

[9] Członka S., Bertino M.F., Strzelec K., Strąkowska A., Masłowski M. (2018). Rigid polyurethane foams reinforced with solid waste generated in leather industry. Polym. Test..

[10] Kurańska M., Benes H., Polaczek K., Trhlikova O., Walterova Z., Prociak A. (2019). Effect of homogeneous catalysts on ring opening reactions of epoxidized cooking oils. J. Clean. Prod..

[11] Fridrihsone A., Romagnoli F., Kirsanovs V., Cabulis U. (2020). Life Cycle Assessment of vegetable oil based polyols for polyurethane production. J. Clean. Prod..

[12] Kurańska M., Banaś J., Polaczek K., Banaś M., Prociak A., Kuc J., Uram K., Lubera T.J. (2019). Evaluation of application potential of used cooking oils in the synthesis of polyol compounds. Environ. Chem. Eng..

[13] Zhang J., Hori N., Takemura A. (2020). Influence of NCO/OH ratio on preparation of four agricultural wastes liquefied polyols based polyurethane foams. Polym. Degrad. Stab..

[14] Borowicz M., Paciorek-Sadowska J., Isbrandt M., Grzybowski Ł., Czupryński B. (2019). Glycerolysis of Poly(lactic acid) as a Way to Extend the “Life Cycle” of This Material. Polymers.

[15] Cateto C.A., Barreiro M.F. (2009). Optimization Study of Lignin Oxypropylation in View of the Preparation of Polyurethane Rigid Foams. Ind. Eng. Chem. Res..

[16] Kurańska M., Pinto J.A., Salach K., Barreiro M.F., Prociak A. (2020). Synthesis of thermal insulating polyurethane foams from lignin and rapeseed based polyols: A comparative study. Ind. Crop. Prod..

[17] Hejna A., Kirpluks M., Kosmela P., Cabulis U., Haponiuk J., Piszczyk Ł. (2017). The influence of crude glycerol and castor oil-based polyol on the structure and performance of rigid polyurethane-polyisocyanurate foams. Ind. Crop. Prod..

[18] Paruzel A., Michałowski S., Hodan J., Horak P., Prociak A., Benes H. (2017). Rigid Polyurethane Foam Fabrication Using Medium Chain Glycerides of Coconut Oil and Plastics from End-of-Life Vehicles. ACS Sustain. Chem. Eng..

[19] Ji D., Fang Z., He W., Luo Z., Jiang X., Wang T., Guo K. (2015). Polyurethane rigid foams formed from different soy-based polyols by the ring opening of epoxidised soybean oil with methanol, phenol, and cyclohexanol. Ind. Crop. Prod..

[20] Jiao L., Xiao H., Wang Q., Sun J. (2013). Thermal degradation characteristics of rigid polyurethane foam and the volatile products analysis with TG-FTIR-MS. Polym. Degrad. Stab..

[21] Bryśkiewicz A., Zieleniewska M., Przyjemska K., Chojnacki P., Ryszkowska J. (2016). Modification of flexible polyurethane foams by the addition of natural origin fillers. Polym. Degrad. Stab..

